# Retrospective Survey and Analysis of Anaesthesia and Outcomes in Paediatric Cleft Lip or Palate Surgery in a Tertiary Care Center, Portugal

**DOI:** 10.7759/cureus.34711

**Published:** 2023-02-07

**Authors:** Daniel Teles, Diana Rodrigues, Marisa Barros, Ana Silva, João Maia, Amélia Ferreira

**Affiliations:** 1 Anaesthesiology, Centro Hospitalar Universitário São João, Porto, PRT

**Keywords:** cleft lip, cleft palate, airway managment, anaesthesia, perioperative complications

## Abstract

Introduction: Orofacial clefts are the most common craniofacial abnormalities, affecting approximately one in 700 newborns each year. The anaesthetic management of these patients is challenging, including difficulties in airway approach and respiratory complications that have direct implications in the final outcome.

Aim: The present study aimed to characterize the anesthetic approach to paediatric patients undergoing cleft palate or lip surgical repair and review the perioperative anesthetic complications in a tertiary Portuguese hospital.

Methods: Data were collected from a retrospective review of the patient records which included anaesthesia perioperative notes of paediatric patients submitted to cleft surgery repair during a five-year period (2016 to 2021). Demographic, pre-anaesthetic characteristics, anaesthetic management and perioperative complications were recorded.

Results: A total of 102 patients were included, with a median age of 1.5 years. Congenital syndromes were present in 14 (13.7%) of the children included. Inhalational induction of anaesthesia with sevoflurane was the preferred approach in 86 cases (84.3%), with neuromuscular blockade being used in 59 cases (57.8%). Intubation was achieved at first attempt in 91 (89.2%) cases with four (3.9%) patients needing three or more attempts. Intraoperative respiratory-related complications were the most frequent, occurring in 22 (21.6%) cases. These include multiple attempts to intubation, desaturation due to bronchospasm or laryngospasm. The average length of stay was two days. Postoperative complications were recorded in 17 (16.7%) of patients.

Discussion: The predominance of airway and respiratory complications occurring in cleft is consistent with previous studies. Care must be taken in order to avoid such complications in the perioperative period by following protocols, having skilled personnel, appropriate monitoring equipment and airway devices available during cleft surgeries to minimise morbidity.

## Introduction

Worldwide, cleft lip and/or palate are the most common congenital craniofacial anomalies in children, affecting approximately one in 700 newborns each year [1]. This occurs due to the failure of the fusion of the nasal and maxillary processes with the palatine shelves, which are formed during the eighth week of the embryonic period [2]. Many classifications have been described, but essentially the cleft can involve the lip, alveolus (gum), hard palate and/or soft palate and can be complete or incomplete, unilateral or bilateral. Cleft lip and palate are also associated with an increased incidence of other congenital abnormalities. The most well‑known are Pierre Robin, Treacher Collins and Goldenhar syndromes [2,3]. Furthermore, infants with these facial deformities are usually associated with abnormal dentition, hearing defects, recurrent ear/upper respiratory tract infections, congenital heart disease, pulmonary aspiration and poor nutrition [2].

Patients proposed for cleft surgical repair are at an increased risk of perioperative complications [1-4]. Main anesthetic complications are respiratory and related to the airway, which may include a difficult airway, inadvertent extubation, kinking of endotracheal tube, aspiration of blood and secretions, laryngospasm or bronchospasm, which can have implications in the final outcome. There is a wide range of common factors associated with these complications, including infants or younger children and concomitant preoperative respiratory symptoms [2,4,5].

The present study aims to characterize the anaesthetic approach of paediatric patients undergoing cleft lip or palate surgery repair, review the perioperative anaesthetic complications, in addition to assessing the relationship between patient or anaesthetic technique and the occurrence of perioperative complications in a tertiary Portuguese teaching hospital.

## Materials and methods

Following Institutional Ethical Committee approval from Centro Hospitalar Universitário de São João (number 295/21), data were retrospectively collected from reviewing the electronic clinical and anaesthetic records of 102 patients submitted to cleft surgical repair during a five-year period, between 2016 and 2021 in a single paediatric centre in Porto, Portugal. The inclusion criteria were: patients under 18 years of age, diagnosis of cleft lip or palate, submitted to cleft surgical repair and availability of electronic anaesthetic records.

Recorded data included a demographic and clinical profile (age, sex, American Society of Anesthesiologists [ASA] physical status score, type of cleft, associated syndromes and other comorbidities), the anaesthetic approach (anesthetic technique, airway management), perioperative complications (respiratory, cardiovascular) and postoperative evolution (length of hospital stay, intensive care unit admission).

For the purpose of obtaining descriptive statistics, patients were divided into four age groups (less than one year, one to three years, four to 10 years and 11 to 17 years).

Statistical analysis

Data were analysed using the SPSS statistics for Windows version 27.0.1 (IBM Corp., Armonk, NY, USA). Descriptive statistics with numerical data were expressed as median and interquartile range (IQR) while for qualitative variables, absolute (n) and relative frequencies (%) were calculated. Tests of significance (Mann-Whitney U, Chi-square and Fisher’s exact test) were used whenever appropriate. A p-value < 0.05 was considered statistically significant.

## Results

Patient characteristics

From 2016 to 2021, a total of 102 patients were included in the present study. The median age at the time of surgery was 1.5 years, and 35.3% of patients were less than one year old. Fifty-two percent of the patients were male. Most of the aforementioned patients presented with lip and palate cleft (58.8%). There were 44 patients with associated comorbidities (43%), 14 of which (13.7%) had associated clinical syndromes: seven Pierre Robin syndrome, three Stickler syndrome, one Goldenhar syndrome, one Klinefelter syndrome, one distal trisomy 6q syndrome and one polymalformative syndrome (congenital heart disease, bilateral hallux polydactyly, appendix of the fifth finger of the left hand and lip and palate cleft). The majority of the patients (86.3%) were classified as either ASA physical status score I or II (Table 1).

**Table 1:** Patient characteristics ASA: American Society of Anesthesiologists

Median age in years, (Interquartile range)	1.5 (0.7-11.1)
<1 year, n (%)	36 (35.3%)
1 - 3 years, n (%)	28 (27.5%)
4 - 10 years, n (%)	12 (11.8%)
11 - 17 years, n (%)	26 (25.5%)
Sex	
Male, n (%)	53 (52%)
Female, n (%)	49 (48%)
Type of cleft	
Cleft lip, n (%)	14 (13.7%)
Cleft palate, n (%)	28 (27.5%)
Cleft lip and palate, n (%)	60 (58.8%)
Associated Syndrome	
Yes, n (%)	14 (13.7%)
No, n (%)	88 (86.3%)
ASA Physical Status	
I, n (%)	30 (29.4%)
II, n (%)	58 (56.9%)
III, n (%)	14 (13.7%)

Anaesthetic approach

Regarding the anaesthetic technique, all patients were submitted to general anaesthesia, which was combined with infraorbital block in 12.7% of the cases of cleft lip surgical repair. Inhalation induction was the technique of choice in 84.3% of the patients. Patients submitted to inhalation induction were statistically significantly younger than patients that had an intravenous induction (mean age 1.3 years vs 13 years, p<0.001). Neuromuscular blocking agents were administered in 57.8% of cases. Opioids were used in the intraoperative period as part of a balanced anaesthesia in 94% of the patients. Furthermore, dexamethasone was administered intraoperatively in 92.2% of patients (dose 0.1 - 0.15mg/Kg) mainly as a prophylaxis for postoperative nausea and vomiting. Hydrocortisone was used in 12.7% of the cases, always added to dexamethasone, usually as a therapy towards a perceived greater probability of consequences from airway and respiratory system manipulation from either anaesthesia or surgery.

Direct laryngoscopy was the airway approach technique used in most of the cases (87.3%). Intubation at the first attempt was achieved in 89.2% of cases. However, in 3.9% of patients there were at least three intubation attempts. A fiberoptic intubation was performed in a Pierre Robin syndrome patient after three attempts of direct laryngoscopy. Orotracheal reinforced tubes (ETT) were placed in 49.0% of cases, while oral Ring-Adair-Elwyn (RAE) tubes were chosen in 45.1% of patients and only one patient (1%) was intubated using a nasal RAE tube.

Complications

Complications in the intraoperative period were apparent in 39% of the patients and include both respiratory and cardiovascular complications.

There were 22 patients (21.6%) with intraoperative respiratory complications, including multiple airway attempts, transitory hypoxemia, laryngospasm and bronchospasm. This rate is similar to the subgroup of syndromic patients (21.4%). However, in the concrete case of Pierre Robin syndrome patients, intraoperative respiratory complication were registered 42.9% of the cases. Among the 22 patients with respiratory complications, 13 were aged up to one year, and eight were aged between one and three years, conferring a higher rate of intraoperative respiratory complications in these particular age groups, 36.1% and 28.6%, respectively. A statistically significant difference was found in the age of patients with and without intraoperative respiratory complications (median age one year vs 2.2 years, p<0.001). However, statistically significant differences in intraoperative respiratory complications rate according to induction anaesthesia technique (p=0.091) have not been found.

Intraoperative cardiovascular complications were registered in 18.6% of patients and included episodes of brady/tachycardia or hypo/hypertension (Table 2).

**Table 2:** Anesthetic approach and intraoperative complications

Anesthetic Approach	
Inhalation induction, n (%)	86 (84.3%)
Intravenous induction, n (%)	16 (15.7%)
Neuromuscular block, n (%)	59 (57.8%)
Intraoperative opioid, n (%)	96 (94.1%)
Infraorbital block, n (%)	13 (12.7%)
Intubation technique	
Direct laryngoscopy, n (%)	89 (87.3%)
Videolaryngoscopy, n (%)	4 (3.9%)
Fibroscopy, n (%)	1 (1.0%)
Missing, n (%)	8 (7.8%)
Intubation attempts	
1, n (%)	91 (89.2%)
2, n (%)	7 (6.9%)
≥ 3, n (%)	4 (3.9%)
Endotracheal tube	
ETT, n (%)	50 (49.0%)
Oral RAE, n (%)	46 (45.1%)
Nasal RAE, n (%)	1 (1.0%)
Missing, n (%)	5 (4.9%)
Corticosteroid	
Dexamethasone, n (%)	94 (92.2%)
Hydrocortisone, n (%)	13 (12.7%)
Intraoperative complications	
Respiratory, n (%)	22 (21.6%)
Cardiovascular, n (%)	19 (18.6%)

Postoperative period

The mean length of hospital stay was two days. The majority of patients (79.4%) were discharged up to two days postoperatively and the most prolonged hospital stay was 10 days. Intensive care unit admission occurred in a total of three (2.9%) patients.

Postoperative complications occurred in 17 patients (16.7%), including seven cases of postoperative fever, five cases of facial swelling, two cases of upper airway infections, one case requiring re-intervention, one case of uncontrolled postoperative pain and finally, one postoperative death due to massive bleeding. As such, the perioperative mortality rate was 1%.

The length of hospital stay was longer in patients with postoperative complications (median 3.0 vs 1.0 days, p=0.002), with no difference in patients who presented intraoperative respiratory complications (p=0.927) (Table 3).

**Table 3:** Postoperative characterization

Length of hospital stay in days, (Interquartile range)	2.0 (1-2)
0-2 days, n (%)	81 (79.4%)
3-5 days, n (%)	18 (17.6%)
6-8 days, n (%)	2 (2.0%)
9-10 days, n (%)	1 (1.0%)
Intensive care unit admission, n (%)	3 (2.9%)
Postoperative complications, n (%)	17 (16.7%)
Postoperative death, n (%)	1 (1.0%)

## Discussion

The present study may be considered as being a pioneer study regarding the anaesthetic approach in pediatric patients undergoing cleft lip or palate surgery repair in a Portuguese paediatric surgical centre.

In the cohort of patients studied, the high prevalence of associated diseases (43%) is in line with previous studies. The complexity of these patients is further illustrated by the significant proportion of patients with associated clinical syndromes (13.7%) [2].

Anesthetic approach

Regarding the anaesthetic plan, there was no established institutional protocol and the choice of the technique was made on an individual base, most commonly led by an anaesthesiologist with experience in peadiatric anaesthesia.

We found that the inhalation induction of anaesthesia with sevoflurane was performed in the majority of the cases, and furthermore that it was significantly more frequently used in younger patients when compared to older ones. The preponderance of this technique in younger patients is due to the current practice in our institution of establishing IV access after the inhalation induction when a child is unable to cooperate preoperatively. Another significant reason for the use of this technique is the ability to maintain spontaneous ventilation in the presence of a difficult airway.

The use of muscle relaxation allows perfectly controlled ventilation which may reduce blood loss secondary to tighter control of partial pressure of carbon dioxide (PaCO2). Spontaneous ventilation is another safe and acceptable technique for these procedures. In our sample, neuromuscular blocking agents were administered in 57.8% of the cases with rocuronium being used as the agent of choice. No associations were found between the use of neuromuscular blocking agents and the presence of an associated syndrome or difficult airway. Moreover, dexamethasone, which has both antiemetic and anti-inflammatory effects, was the preferred glucocorticoid used in these patients.

The anatomical defects of the cleft lip or palate commonly impact the risk of a difficult laryngoscopy and intubation as the cleft alveolus, protruding maxilla and high vaulted arch cause a challenging placement of the laryngoscope blade due to a lack of support which alters the line of vision [5]. Besides this, it should be noted that are also some airway features associated with some of the patients presented for surgery, such as retrognathias, which can be more relevant than the actual anatomical defects themselves. 

The ideal airway device for cleft repair, allowing for optimal surgical access, is a preformed oral RAE tracheal or a reinforced tube. Both options are associated with lower incidence of intraoperative kinking [1], which were also the most common in this type of procedure in our institution.

The present study determined a 10.8 % incidence of more than one attempt at intubation. Considering that the first attempt at intubation is usually performed by an anaesthesia trainee in our centre, this finding is in accordance with previous studies with documented incidence of difficult airway ranging from 4.7% to 8.4% [5-7].

There has been a tendency for increased availability in our centre of videolaryngoscopy and fibroscopic devices, which may play an increasingly important role in the primary airway approach of these patients in the present and future.

Complications

There is a high variability of recorded complications in the literature, with incidences ranging from 8.9% to 58% [3,5,8-11]. The results of this study are within this range (39%). The huge difference in this range can be explained by the difficulty of extracting coherently classified events, which inevitably has a significant limiting impact in a retrospective study of this kind.

According to the literature, the majority of anaesthetic complications related to craniofacial anomalies are airway and respiratory complications such as difficult intubation, endotracheal tube-related problems, laryngospasm or bronchospasm and desaturations [10]. Yet, the most common complications found in the present study were mostly respiratory complications, in 21.6% of the cases. It should be noted that there is a high variability of the incidence of respiratory complications depending on the criteria for inclusion and exclusion of certain events [2,11-13]. A great proportion of respiratory complications can be explained by the mere cohort selected, since bronchospasm or laryngospasm are found to occur in up to 7% of paediatric patients during general anaesthesia [14,15].

The cohort of this study presented an association between age and airway complications, with a higher proportion of complications in younger infants less than one year, which is coherent with the findings of the literature of higher incidence of intraoperative adverse events in this age group due to the increased reactivity of the tracheobronchial tree in this subgroup [15-17].

No statistical differences in the complications rate of patients with clinical syndromes were found, although the higher incidence of intraoperative respiratory complications in Pierre Robin syndrome patients (42.9%) should be pointed out.

In addition, statistically significant differences in intraoperative respiratory complications rate according to induction anaesthetic technique as described in other studies were not confirmed by the present study [5].

Reports of postoperative complications are often scarce. A global incidence of complications of 16.7% was found in this study. The mortality rate noted in other studies is very low and usually described as a consequence of severe preexisting cardiovascular comorbidities [2,10,13]. Yet, in the case of this particular study, it should be noted that there was a sole occurrence of one case of postoperative death. It was due to hemorrhagic shock resulting from massive postoperative surgical wound bleeding, which was impossible to control surgically.

Recommendations 

The avoidance of these complications and minimization of morbidity rates is achieved by implementation of protocols in the perioperative period to improve standards of care, including a comprehensive preoperative anaesthetic assessment and planning of the postoperative disposition and anticipated complications [18]. 

Patients with clefts should be managed by skilled personnel, in specialist paediatric centres, with the appropriate monitoring and airway equipment, including indirect laryngoscopy devices which, as stated above, can be of special importance in patients where a difficult airway might be expected, such as patients less than six months of age, bilateral cleft lips, and retrognathia [6]. 

The destination and the time of the postoperative period have to take into account the need for surveillance of postoperative complications, particularly in patients proposed for primary cleft palate repair as they are at higher risk of postoperative bleeding and airway obstruction. Depending on the surgery, intraoperative status, and comorbidities, this can be achieved in a standard ward or there may be the need for admission to a paediatric intensive care unit [18].

Limitations

The findings of this study should be interpreted in the context of the study design and its limitations. As it is a retrospective study, there may be a lack of data regarding the perioperative events. It is possible that there is an under-reporting of some lighter complications such as transient desaturations, hypothermia or mild hypotension as these did not amount to considerable implications in the perioperative pathway. Given the broad potential impact of critical events on these patients, it is our firm belief that when critical incidents occur, they must be recorded in an appropriate manner so as to allow researchers to then determine the corresponding explanations and associated factors with the events in question and, ultimately, to contribute to the prevention of any further occurrences of this nature.

Another limitation of this study was that the level of experience of the anaesthesiologist who performed tracheal intubation was not recorded, which is important for the interpretation of data regarding intubation attempts. This is because, as previously mentioned, this study was conducted in a teaching hospital where residents often perform the first attempt of intubation.

## Conclusions

Young patients in need of a primary repair of a cleft lip or palate present a significant anaesthetic challenge. In this context, it is essential to anticipate factors that may cause perioperative complications and challenging airway implications to improve patient safety in both patients and surgical procedures.

The present study regarding the anaesthetic approach and perioperative complications in paediatric patients undergoing cleft surgery repair in a Portuguese paediatric surgical centre, has allowed us to clearly demonstrate that the current practice is in conformity with other specialised centres in terms of having a similar incidence of complications and opens the opportunity for continuous improvement in the perioperative care of these patients.
